# Comparison of Devices Used for Stent-Assisted Coiling of Intracranial Aneurysms

**DOI:** 10.1371/journal.pone.0024875

**Published:** 2011-09-22

**Authors:** Benjamin Izar, Ansaar Rai, Karthikram Raghuram, Jill Rotruck, Jeffrey Carpenter

**Affiliations:** 1 Department of Medicine, Massachusetts General Hospital, Boston, Massachusetts, United States of America; 2 Department of Radiology, West Virginia University, Morgantown, West Virginia, United States of America; 3 School of Medicine, West Virginia University, Morgantown, West Virginia, United States of America; Stanford University Medical Center, United States of America

## Abstract

**Introduction:**

Two self-expandable stents, the Neuroform and the Enterprise stent, are widely used for stent-assisted coiling (SAC) of complex shaped intracranial aneurysms. However, comparative knowledge about technical feasibility, peri- and post-procedural morbidity and mortality, packing densities as well as follow-up data is limited.

**Material and Methods:**

We conducted a retrospective study to investigate differences in aneurysms stented with the Enterprise or Neuroform stents. Angiographic follow-up (mean 19.42 months) was available in 72.6% (61/84) of aneurysms treated with stent-assisted coiling. We further sought to compare stent-assisted coiling to a matched patient population with aneurysms treated by conventional coil embolization.

**Results:**

The stenting success rate of the Enterprise was higher compared to the Neuroform stent (46/48 and 42/51, respectively). In 5 of 9 cases in which the Neuroform stent was not navigable to the landing zone, we successfully deployed an Enterprise stent instead. Eventually, 42 aneurysms were coiled after stenting in each group. We observed no significant differences in peri-procedural complication rate, post-procedural hospital stay, packing density, recurrence rate or number of in-stent stenosis. Strikingly, 36.1% of followed aneurysms in the SAC group showed progressive occlusion on angiographic follow-up imaging. The packing density was significantly higher in aneurysms treated by SAC as compared to conventionally coiled aneurysms, while recanalization rate was significantly lower in the SAC group.

**Conclusion:**

The procedural success rate is higher using the Enterprise, but otherwise both stents exhibited similar characteristics. Lower recurrence frequency and complication rates comparable to conventional coil embolization emphasize the importance of stent-assisted coiling in the treatment of complex aneurysms. Progressive occlusion on angiographic follow-up was a distinct and frequent observation in the SAC group and may in part be due to flow diversion.

## Introduction

Stent-assisted coiling (SAC) allows endovascular treatment of complex shaped and wide necked intracranial aneurysms, which are challenging for conventional coil embolization (CCE). Currently, two stents are approved for SAC in the USA, the Enterprise Vascular Reconstruction device (Enterprise, EP) (Codman Neurovascular/Johnson & Johnson) and the Neuroform stent (NF) (Boston Scientific).

Prior reports have tabulated the procedural feasibility, morbidity and mortality associated with each stent individually. There is a wide range of results reported for each stent regarding several outcomes, including stenting success rate, initial grade of occlusion and complication rates [4], [8], [9], [13], [18]. An interpretation of data gathered from different centers using either stent or differences between the two available stents may therefore be difficult. Although both stents are frequently used for SAC, mid-term results associated with either stent are restricted to a relatively small number of studies with angiographic follow-up durations of up to 6.9 months for the EP [7], [8] and 4.6 to 12.1 [9]–[14] months for the NF, and long-term follow-up experience is scarce [15], [16].

The packing density is defined as the ratio between the volume of coils inserted into the aneurysm and the aneurysm volume. In conventional coil embolization a high packing density is associated with lower recurrence and retreatment rates [1], [2]. Experimental aneurysm embolization with Enterprise stent assistance was shown to achieve higher packing densities than aneurysms coiled without prior stent placement [3]. However, it is unclear whether SAC provides a clinical advantage with respect to achieving higher packing densities and how packing densities influence recurrence rates.

Previous studies reported that aneurysms with remaining flow into the aneurysm sac after initial treatment were found to have higher occlusion grades on follow-up imaging. This process, also known as progressive occlusion is associated with reduced blood flow in the sac of aneurysms as observed in studies using flow diverter stents [5]. Similar observations were made for aneurysms treated with the Neuroform stent indicating that flow diversion may also contribute to delayed aneurysm occlusion in SAC in the mid-term follow-up [6].

The goal of this single-center study was to evaluate several parameters in the comparison of the Neuroform and Enterprise stents in stent-assisted coiling and related midterm follow-up findings. We further put these results into the context of conventional coil embolization of intracranial aneurysms.

## Materials and Methods

### Patients and aneurysm treatment

Under institutional review board approval, we retrospectively analyzed the imaging and medical charts of 356 consecutive patients with 385 aneurysms coiled between November 2001 and February 2010. 270 patients harboring 292 aneurysms were treated with conventional coil embolization. 86 patients with 93 aneurysms were intended to be treated by stent-assisted coiling of which 84 aneurysms (n = 42 using the Neuroform and n = 42 using the Enterprise stent) were coiled after successful stent-placement. Five aneurysms, which initially failed treatment with Neuroform (NF), were eventually stented using the Enterprise stent (EP). Stent-assisted coiling was performed in aneurysms with a dome-to-neck ratio <2 or a neck diameter >4 mm and in complex shaped aneurysms that were not approachable by conventional coil embolization. Aneurysms were either coiled with the jailing technique (with NF or EP) or coiling was performed after placement of the stent by taking advantage of the open cell design of Neuroform stents.

### Surgical procedure and perioperative medication

Coiling in all patients was performed under general anesthesia approach in a sterile environment using a common femoral artery approach (except for two brachial) and full anticoagulation with heparin. Heparin anticoagulation was delayed until placement of the initial coil in ruptured aneurysms. The activated clotting time was kept at 250 or higher during all procedures. In unruptured aneurysms treated with SAC, the patients received a dual anti-platelet therapy, including 75 mg of clopidogrel and 325 mg of aspirin for 5 days before stent placement, and dual anti-platelet therapy for 6 months following the procedure. In ruptured aneurysms treated with SAC, the patients received a weight-adjusted bolus dose of abxicimab (ReoPro, 0.25 mg/kg intravenous bolus) at the time of stent deployment followed by 150 mg of clopidogrel and 650 mg of aspirin 12 hours later, and followed by 75 mg of clopidogrel and 325 mg of aspirin daily. Ventricular drains were placed prior to SAC interventions in the cases of ruptured aneurysms.

### Data collection and statistical analysis

In addition to clinical presentation and patient demographics, data was collected for aneurysm size, location, shape (ellipsoid vs. spherical), orientation (sidewall vs. bifurcation), morphology (fusiform vs. saccular), packing density, rupture status and periprocedural morbidity and mortality. In order to put the SAC results into the context of conventional coil embolization, we used the optmatch package (version 0.7–1) of the R tool (version 2.12.1, http://cran.r-project.org) to match patients with aneurysms treated by conventional coil embolization to patients treated by SAC. The aneurysms were matched regarding patient age and gender, aneurysm size, localization, orientation as well as the aneurysm rupture status. Pearson's Chi square and Fisher's exact test were determined for demographic and contingency differentiation. Student's *t* test was used to determine statistical significance in continuous scaled parameters, including age, packing density, post-procedural hospital stay and aneurysm size. A p<0.05 was considered statistically significant.

### SAC procedure success evaluation

All treated aneurysms were graded independently by two experienced interventional neuroradiologists using several views for each treated aneurysm, including 3-dimensional angiography. Assessment of the degree of aneurysm occlusion was evaluated as complete occlusion (OG1), neck remnant (OG2) and residual filing of the aneurysm sac (OG3) [2]. Angiograms were further assessed for in-stent stenosis or thrombosis and stent migration. Inter-rater agreement was indicated by the Cohen's Kappa (K).

### Packing density

Aneurysm packing percentage was calculated as coil volume divided by aneurysm volume. Coil volume was calculated by summing individual coil volumes as indicated by their manufacturers. Aneurysm dimensions were measured by 3-dimensional images derived from rotational angiography. Because aneurysm volume exhibit meaningful variation depending on the calculation method, we classified aneurysms based on their shape into either ellipsoids or spheroids. Aneurysms with a dome-to-neck length ( = height)>1.5× diameter or diameter >1.5× height were designated as ellipsoids, all others were designated as spheres.

### Follow-up protocol

Immediate post-treatment angiographic images were compared to similar projection follow-up images. A conventional angiography was obtained in all but five cases where magnetic resonance angiography or computed tomography angiography of diagnostic quality was obtained. The standard follow-up protocol included an angiography within 6 months after endovascular treatment, a second angiography after 1 year, and a third and fourth being performed 2 and 3 years post-treatment, respectively. In the absence of recanalization, further follow-up occurred in 2 year intervals. In the case of recurrence or additional untreated aneurysms, patients were retreated/treated when necessary and followed according to the same scheme. Patients were assessed immediately after the procedure and a neurological exam was performed at follow-up visits prior to imaging. Major neurological complications were defined as Glascow Outcome Score (GOS)<5.

## Results

### Characteristics of treated aneurysms

93 aneurysms in 86 patients qualified for treatment by SAC. Of the aneurysms intended to be treated by SAC, 84 (42 EP and 42 NF, respectively) were successfully stented and subsequently coil embolized (*Table 1*). 67% of the aneurysms were located in the anterior and 33% in the posterior circulation (*Table 2*). The mean aneurysm size was 7.43 mm (±3.13) in the EP and 7.6 mm (±2.35) in the NF group. 13 aneurysms stented by EP had fusiform morphology and 29 were saccular, while 13 were fusiform and 29 had saccular morphology in the NF group. A total of 7 ruptured aneurysms (8.33%), 3 in the EP group and 4 in the NF group were treated by SAC). More sidewall aneurysms (29 EP and 33 NF) than bifurcation aneurysms (13 EP and 9 NF) were treated by SAC.

**Table 1:** Baseline Demographics of aneurysms treated by stent-assisted coiling using the Enterprise or Neuroform stent.

	Enterprise	Neuroform	T-Test (p-value)	Likelihood ratio (p-value)	OR	95% CI
**Age (years±SD)**	55.2±11.65	56.7±11.84	0.5377			
**Gender (f/m)**	38/4	37/5		1	0.772	0.106–4.91
**Mean max dimension (mm±SD)**	7.43±3.13	7.6±2.35	0.7812			
**localization (ant/post)**	28/14	29/13		1	0.898	0.324–2.475
**Orientation (SW/Bifurcation)**	29/13	33/9		0.457	0.612	0.199–1.811
**Morphology (saccular/fusiform)**	29/13	29/13		1	1	0.358–2.796
**Shape (sphere/ellipsoid)**	36/6	33/9		0.57	1.627	0.459–6.2
**Unruptured/Ruptured**	39/3	38/4		1	1	0.233–4.293
**Packing density (%±SD)**	35.99±22.96	37.96±22.9	0.8096			
**Recurrence (present/absent)**	4/26	3/28		0.707	1.427	0.218–10.685

Numbers refer to aneurysms that were eventually coiled by stent assistance (n = 42 with Neuroform and n = 42 with Enterprise). Abbreviations: f (female), m (male), mm (millimeters), ant (anterior circulation), post (posterior circulation), SW (side wall), SD (standard deviation), OR (odds ratio), CI (confidence interval).

**Table 2:** Location and mean maximum diameter of aneurysms stented using either the Enterprise or Neuroform stent and subsequently treated by coiling.

	SAC (n = )	Enterprise (n = )	Mean dimension (mm)	Neuroform (n = )	Mean dimension (mm)
**ICA cavernouse segment**	9	4	7,75	5	8,28
**ICA ophthalmic segment**	36	18	7,49	18	7,73
**ICA supraclinoid segment**	5	3	8,83	2	8,2
**MCA**	1	0		1	4,5
**AC**	4	3	7,8	1	5
**PC**	2	0		2	8,35
**Basilar apex**	18	10	7,37	8	7,54
**Basilar trunk**	1	0		1	6
**Vertebral artery**	3	2	8,1	1	7
**SCA**	2	2	5,8	0	
**PICA**	3	0		3	7,03
**Total (n = )**	84	42		42	

Abbreviations: SAC (stent-assisted coiling), n (number of aneurysms), mm (millimeters), ICA (internal carotid artery), MCA (middle cerebral artery), AC (anterior communicating artery), PC (posterior communicating artery), SCA (superior cerebellar artery) and PICA (posterior inferior cerebellar artery).

The majority of aneurysms were discovered incidentally (48/90) followed by aneurysms that were previously discovered, and aneurysms that became clinically apparent, most frequently due to cranial nerve compression symptoms and headache.

### Procedural feasibility of stents

**Figure 1:**
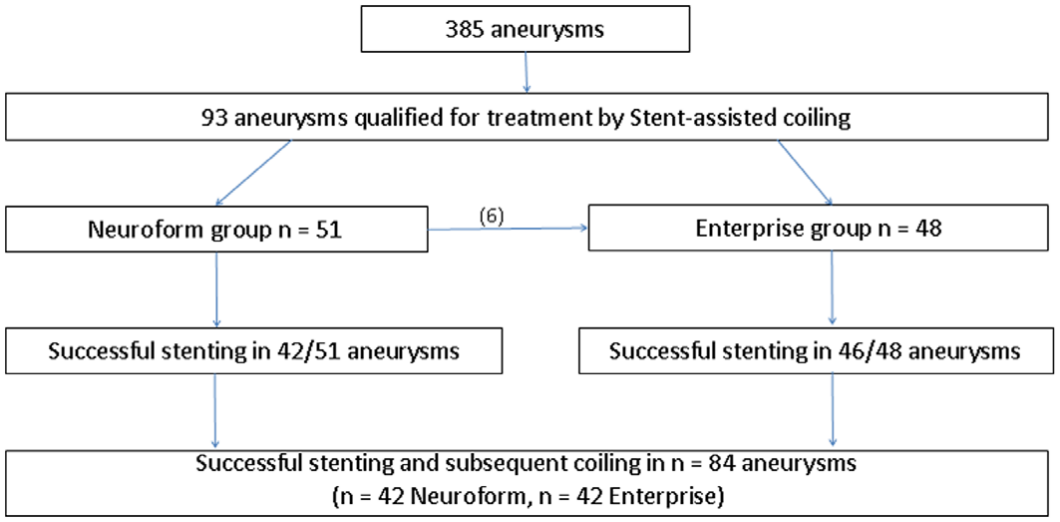
Flow chart of aneurysms treated by stent-assisted coiling.

Navigation and subsequent deployment of the Enterprise stent was significantly more often successful as compared to using the Neuroform stent (p = 0.0329) (*Figure 1*).

Of the 51 aneurysms intended to be treated by SAC using the NF, 42 (82.3%) were successfully stented and coiled by the jailed-catheter technique in all but in one cases. In this patient, the stent was deployed over the aneurysm neck after a previously placed coil prolapsed into the parent artery. In two patients, a single stent sufficiently covered the neck of 2 aneurysms. In 8 cases the NF stent could not be navigated to the desired landing zone and had to be removed. Six of these were located in the anterior circulation (2 ophthalmic segment ICA, 1 terminal ICA, 1 posterior communicating artery, 1 supraclinoid ICA and 1 in the cavernous ICA) and 2 in the posterior circulation (1 posterior cerebral artery, and 1 in the vertebrobasilar junction). Inability to navigate the NF to the landing zone was associated with tortuosity of the vasculature. Furthermore, we had one case, in which a Neuroform stent expanded unplanned in the ICA.

In 6 cases where the NF was not navigable, we attempted to place an EP instead. The EP was successfully navigated in all 6 cases and deployed in 5 cases. Subsequent coiling was performed without complications. In the remaining case, the operator decided not to deploy the EP stent due to high resistance.

Of the 48 aneurysms, including the above mentioned 6 cases, intended to be treated with the EP, the stent was successfully navigated and deployed in 46 (95.8%). In two patients, one EP covered two adjacent aneurysms and in an 81 year old patient with a ruptured side-wall basilar aneurysm, two stents were used to cover one aneurysm. In 42 (91.3%) aneurysms subsequent coiling was performed in the same session.

### Procedure related morbidity and mortality

We observed no significant differences in peri-procedural morbidity and mortality between both stents. Procedure related morbidity with long-term neurological sequela (GOS = 3) was 1 (1.9%) in 51 cases in which the NF was placed or intended to be placed. In the EP group, the treatment of 2 (4.2%) aneurysms was associated with procedure related long-term neurological complications (GOS = 4 in both cases). Most minor and major peri-procedural complications observed in this study were related to anti-platelet therapy and thromboembolism (*Table 3*). Minor procedure related events, such as groin hematoma, iliac hematoma and headache occurred in 7 patients (3 in the EP and 4 in the NF group).

**Table 3:** Periprocedural mortality and major complications in patients treated in the by stent-assisted coiling.

Patient age, gender	aneurysm localization	aneurysm size (mm)	shape, status	stent	complication	Treatment	Long-term neurological deficit
**45, f**	basilar apex	2	saccular, ruptured	EP	Rebleeding, hydrocephalus requiring shunt placement	Surgical and medical	absent
**49, f**	basilar apex	29	fusiform	EP	Retroperitoneal hematoma requiring transfusion and ICA dissection requiring stenting	Medical	present (GOS = 4)
**59, m**	vertebral artery	7	fusiform	EP	brachial pseudoaneurysm	Conservative	absent
**41, m**	ophthalmic ICA	9	saccular	EP	Thrombembolism; vision impairment	Medical	present (GOS = 4)
**55, m**	ophthalmic ICA	10	fusiform, ruptured	NF	Cardiac arrest	Medical	absent
**56, f**	ophthalmic ICA	9.4	saccular	NF	Thromboembolism	Medical	present (GOS = 3)
**57, f**	cavernous ICA	25	fusiform	NF	Thromboembolism (small ICA thrombus)	Medical	absent
**77, f**	ophthalmic ICA	6.6	saccular	NF	Epidural hematoma after fall	Conservative	absent
**51, f**	basilar apex	9	fusiform	NF	Retroperitoneal hematoma requiring transfusion	Medical	absent

90 days mortality				Cause			
**71, f**	ophthalmic ICA	5	saccular	NF	Anti-platelet treatment related ICH		
**81, f**	basilar apex	2.5	saccular, ruptured	EP	Hemorrhage (rebleeding)		

Abbreviations: f (female), m (male), mm (millimeters), EP (Enterprise stent), NF (Neuroform stent), ICA (internal carotid artery), ICH (intracerebral hemorrhage), GOS (Glascow Outcome Score).

There was 1 death in the group of patients stented with a NF. The 71 year old patient was treated for a paraophthalmic ICA aneurysm and tolerated the procedure without problems, but suffered from an intraparenchymal hemorrhage while on dual anti-platelet medication. We also observed 1 death in the patient group treated with the EP. The 81 year old patient presented with a ruptured 2.3 mm basilar sidewall aneurysm. During attempted microcatheter access the aneurysm re-ruptured. Although a second stent was deployed across the aneurysm neck, the patient eventually died due to complications of a SAH-related vasospasm.

The procedure related morbidity and mortality of patients treated with SAC was comparable to the matched patient group receiving conventional coil embolization (p>0.05).

In 61 (71.2%) cases, patients were discharged from hospital 1 day after the SAC. Mean post-procedural hospital stay in the NF group was 1.63 days vs. 4.77 days in the EP group, but was not statistically significant (p = 0.0871). Median post-procedural hospital stay was 1 day in both groups.

### Initial angiographic results and delayed aneurysm occlusion in SAC

Angiographic results were evaluated independently by two raters. There was substantial agreement in the reading results (k = 0.652). On the immediate post-treatment angiogram the occlusion grade was as follows for the Enterprise group (*Table 4*): OG1 in 13 (30.95%), OG2 in 16 (38.1%) and in OG3 13 (30.95%) aneurysms; in the Neuroform group we observed OG1 in 18 (42.8%), OG2 in 17 (40.5%) and OG3 in 7 aneurysms (16.67%).

**Table 4:** Initial angiographic outcome of aneurysms coiled with stent-assistance using the Neuroform or Enterprise stent and occlusion grade at final angiographic follow-up.

	Occlusion grade at SAC					Occlusion grade at final follow-up		
	OG1 (n)	OG2 (n)	OG3 (n)	FU available (n)	Mean FU (months)	OG1 (n)	OG2 (n)	OG3 (n)
**Enterprise**	30.95% (13)	38.1% (16)	30.95% (13)	30	14	50% (15)	36.67% (11)	13.3% (4)
**Neuroform**	42.8% (18)	40.5% (17)	16.67% (7)	31	25	48.4% (15)	38.7% (12)	12.9% (4)
**Total SAC**	36.9% (31)	39.3% (33)	23.8% (20)	61	19.42	49.1% (30)	37.7% (23)	13.1% (8)

Abbreviations: OG1 (complete occlusion), OG2 (neck remnant), OG3 (saccular filling), n (number of aneurysms), FU (follow-up), SAC (stent-assisted coiling).

Angiographic follow-up imaging was available for 61 aneurysms (30 EP and 31 NF).

**Figure 2:**
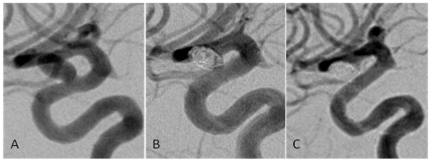
A 45 year old woman presented with double vision and subsequent angiographic imaging revealed bilateral aneurysms of the ICA of which one was located in the right paraophthalmic ICA measuring 4.2 mm. Figure 2A shows the aneurysm in the lateral view before treatment and immediately after stent-assisted coiling using a 4.5×22 mm Enterprise stent (B). Residual flow to the neck of the saccular aneurysm was noted on immediate post-treatment angiography. The 10 months follow-up imaging (C) revealed that the aneurysm underwent progressive occlusion as there was no residual flow visible while the aneurysm displayed satisfactory coil coverage.

**Figure 3:**
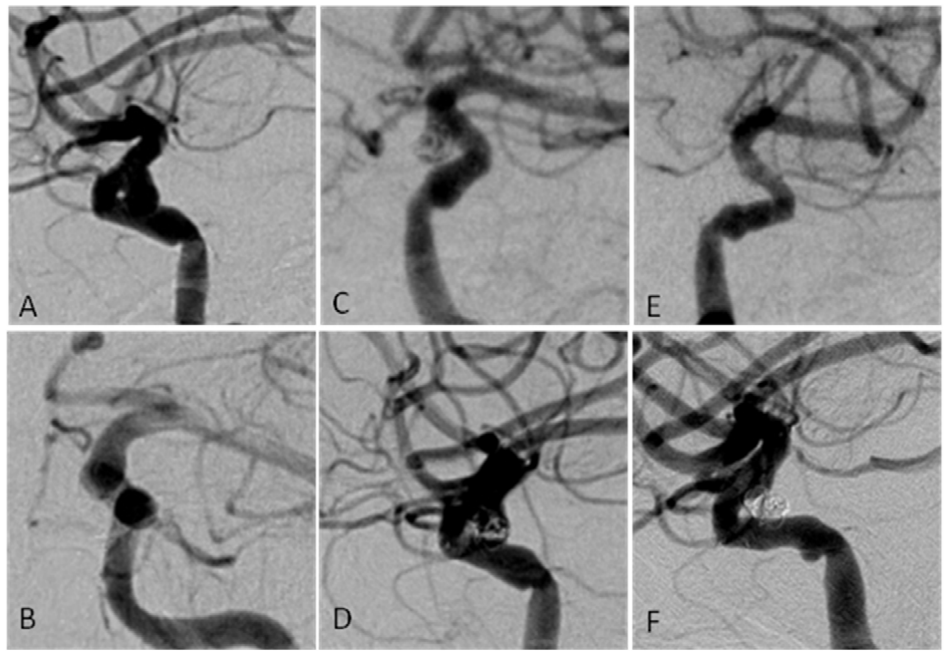
A 64 year old woman presenting with vision impairment was found to have an aneurysm measuring 5 mm that was located in the supraclinoid ICA segment. Pre-treatment angiographic imaging (A and B) revealed a saccular wide-necked aneurysm which was accessible by SAC. A Neuroform stent was successfully navigated, deployed and subsequent coiling was performed. Immediate post-treatment results (C and D) demonstrated only partial occlusion of the aneurysm and flow beyond the aneurysm neck was present. At 6-months follow-up imaging (E) no residual flow into the aneurysm sac was detectable. These results were stable at 18-months follow-up imaging (F) and emphasize the potential of aneurysms to undergo progressive occlusion after treatment by stent-assisted coiling.

The degrees of occlusion in followed aneurysms treated with EP were: OG1 in 15 (50%), OG2 in 11 (37.67%) and OG3 in 3 (13.3%); occlusion grades in the NF group were: OG1 in 15 (48.4%), OG2 in 12 (38.7%) and OG3 in 4 (12.9%) aneurysms. Thus, 22 of 61 (36.1%) followed aneurysms underwent delayed occlusion of the aneurysm sac. The frequency of aneurysms with OG3 flow into the sac decreased from 23.8% on initial angiogram evaluation to 13.1% on the final available follow-up, while the overall frequency of aneurysms with complete sac occlusion increased from 36.9% to 49.1% (*Table 4*). A representative case for each stent is shown in *Figure 2* and *Figure 3*.

Logistic regression analysis revealed that sidewall aneurysms (p = 0.0009) and localization in the anterior circulation (p = 0.0464) favored delayed occlusion of the aneurysm. Progressive occlusion was independent from the aneurysm size and packing density.

Furthermore, we observed partial coil prolapse into the parent vessel in two aneurysms treated with the NF and two in the EP group.

### Packing density

We observed no significant difference in packing density comparing the NF and EP group (p = 0.8096). However, the packing density in the SAC group was clearly higher compared to the CCE group (mean 36.59% vs. 28.58%, p = 0.0189) (*Table 5*). Overall, lower packing density was associated with large aneurysm size (p<0.0001) and ellipsoid aneurysm morphology (p = 0.0056).

**Table 5:** Baseline Demographics of aneurysms treated by stent-assisted coiling and a matched group of aneurysms treated by conventional coil embolization.

	CCE	SAC	T-test (p-value)	Likelihood ratio (p-value)	OR	95% CI
**Age** # **(years±SD)**	55.5±10.76	55.9±10.9	0.8111			
**Gender** # **(f/m)**	75/9	75/9		1	1	0.331–3.019
**Mean max dimension** # **(mm±SD)**	7.49±3.39	7.52±2.75	0.9499			
**localization** # **(ant/post)**	47/37	56/28		0.205	0.637	0.324–1.243
**Orientation** # **(SW/Bifurcation)**	57/27	62/22		0.497	0.75	0.363–1.54
**Shape** # **(sphere/ellipsoid)**	72/12	69/15		0.675	1.30	0.526–3.286
**Ruptured/Unruptured** #	12/72	7/77		0.33	1.827	0.622–5.79
**Packing (**%±**SD)**	29.16±17.46	36.59±22.8	0.0189*			
**Recurrence (present/absent)**	24/60	7/54		0.014*	**3.063**	**1.162–9.112**

Abbreviations: CCE (conventional coil embolization), SAC (stent-assisted coiling), f (female), m (male), mm (millimeters), ant (anterior circulation), post (posterior circulation), SW (side wall), SD (standard deviation), OR (odds ratio), CI (confidence interval).

# indicates patient and aneurysm characteristics that were matched to the SAC group.

### Recurrence rates and time to angiographic recurrence

Aneurysm recanalization rates were comparable for both stents, with 3 occurring in the NF and 4 in the EP group (p = 0.707). Overall, the recurrence rate was lower in aneurysms treated with SAC as compared to the CCE treatment (11.5% vs. 28.5%; p = 0.014). Mean time to angiographic recurrence was 318 days in the CCE group and 208 days in the stented group (p = 0.2408). Anterior circulation aneurysms were associated with a longer time period to recurrence in the CCE group (p = 0.0271), but not in the SAC group (p = 0.7765).

Interestingly, lower packing density was linked to a higher recurrence rate in the CCE group (p = 0.0299), but not in the SAC group (p = 0.4648). The mean packing density determined in recanalized aneurysms was 22.95% in the CCE group vs. 30.53% in the SAC group.

### Follow-up findings

Conventional angiographic follow-up was available for 61 (72.6%) aneurysms treated with SAC with a mean follow-up time of 19.42 months (14 months in the EP, 25 months in the NF group). In the EP group, one asymptomatic stent migration was discovered at 20 month follow-up angiogram for a basilar apex aneurysm. One in-stent stenosis occurred in the EP group discovered 11 weeks after SAC in a 46 year old female with a saccular aneurysms located in the cavernous ICA segment. The stenosis remained stable and the patient was asymptomatic at 6 months follow-up evaluation. Two cases of in-stent stenosis occurred in the NF group. The first resulting in an ipsilateral stroke occurring 6 weeks after SAC in a patient with thrombocytopenia related bleeding that required stoppage of anti-platelet therapy. The second case was discovered 8 weeks after EVT in an asymptomatic patient and was stable as of 55 months follow-up angiography.

## Discussion

Self-expanding stents are frequently used in the coiling of wide-necked and fusiform intracranial aneurysms. However, the clinical impact of available stents and delivery systems remains scarce as indicated by a large meta-analysis [17]. Prior studies have outlined the characteristics of the Neuroform stent, including the stenting success rate, complication rates in ruptured and unruptured aneurysms and in-stent stenosis rates [7], [9], [11]–[13],[18]–[21], while comparatively little published regarding the Enterprise stent [8], [22]. There is limited evidence about comparative benefit of using one of the two FDA approved stents for SAC over the other. Knowledge about long-term results with SAC is scarce [15].

In this study both stented groups were homogenous which allowed a good comparison of the stents. Both stents possessed good to excellent procedural feasibility (82% NF, 96% EP) in close agreement with previously reported success rates of 84 to 90% for the Neuroform and 90 to 100% for the EP [7]–[9], [11]–[14], [18]–[21]. However, the success rate was clearly higher in the Enterprise group and in 5 of 6 cases where the NF could not be placed the EP was successfully deployed.

Both stents were associated with a comparably low peri-procedural complication rate and mortality. In contrast to a large study [4] and despite the more complex morphology of aneurysms treated by SAC, there was no significant difference in peri-procedural morbidity with long-term neurological sequela and mortality between the SAC using either stent and the CCE group. As outlined by the authors, the alarmingly high complication rate of 7.8% in the SAC group may be biased due to the use of balloon mounted stents [4] as compared to self-expandable stents, such as the Neuroform and Enterprise stent. However, the complication rate observed in this study is consistent with other studies ranging from 0 to 16% [8], [9], [11], [12], [14], [18]–[23] determined in studies including ruptured as well as unruptured aneurysms and between 4.9 to 24% [7], [13] in studies with ruptured aneurysms only. Mortality rates in studies including mainly unruptured aneurysms ranged from 0 to 4.6% [8], [9], [12],[18],[19],[21],[22] and 0 to 20% [11], [13], [14] in reports with a high number of or only ruptured aneurysms. Long-term neurological complications occurred in 3.5% of patients treated with SAC and were linked to hemorrhage while on dual anti-platelet therapy and to a lesser degree due to thromboembolism (*Table 3*). The deaths in the SAC group resulted from hemorrhage. One from aneurysm rupture during coiling and the second from a large cortical hematoma that occurred while still on dual anti-platelet therapy. Bleeding episodes occurred with higher frequency early in our experience with SAC and we subsequently reduced the time on dual-anti-platelet therapy from 6 month to 6–12 weeks after SAC. However, bleeding facilitated by dual anti-platelet therapy after stent-assisted treatment remains a concern.

In this study, packing density in the SAC group was higher compared to the CCE group (36.59% vs. 28.58%). Although an in vitro study indicated that packing density was 10.5% higher in aneurysms coiled with stent assistance, this is the first report confirming this result in vivo. Furthermore, we show that not only the EP, which was used in the in vitro study, but also the NF achieved substantially higher packing densities, while no differences in packing densities were observed between both stents.

Recanalization rates using either stent were similarly low. However, we observed significantly lower recurrence rates in the SAC versus CCE group. High packing density was associated with lower recurrence rates in the CCE group, which is consistent with previous reports [1], [4]. However, although packing was higher in the SAC group, it had no impact on recurrence rates in this aneurysm set. This observation was further supported by the observation that the mean packing density in recurrent aneurysms in the CCE was low (23%) [1], but relatively high (31%) in the SAC group. Although controversial, hemodynamic flow changes and redirection of forces induced by placing a stent over the aneurysm neck may have a synergistic effect with coil packing of the aneurysm. This unexpected finding may indicate that high packing densities, although desirable, may not be necessary to adequately occlude some aneurysms with SAC as suggested by previous reports [4], [9], [15].

Initial angiographic results showed that 76.2% of aneurysms treated with stent-assistance had total flow cessation or only minimal flow into the neck (*Table 4*), which is consistent with previous reports [4], [9], [15], [22]. However, 92% of followed aneurysms showed no flow or only minimal into the aneurysms, thus, more than 36% of followed aneurysms showed progressive occlusion. Sidewall orientation, anterior localization, good initial coil coverage influenced delayed occlusion of the aneurysm. Again, progressive sac occlusion was independent from the aneurysm packing density and no major differences were found between both stents. The lower frequency of delayed aneurysm occlusion in the posterior circulation may be related to the fact that most of these aneurysms were bifurcation aneurysms of the basilar apex. The existing stent designs appear to influence bifurcation aneurysm occlusion and thrombosis to a lesser degree.

Both available stents appear to have low rates of in-stent stenosis indicating a good mid-term safety using either stent. Two cases of in-stent stenosis were observed in the NF group, one was detected in the EP group. It is unlikely that differences in length of follow-up for the two stent groups influenced this difference as the stenosis all occurred within 11 weeks following SAC. Spontaneous migration of the EP was previously reported [24] and was seen in one case occurring in a basilar apex aneurysm.

In conclusion, this study shows that the Enterprise stent is more deliverable than the Neuroform stent, but both devices are feasible and safe in use for the treatment of intracranial aneurysms while exhibiting similar immediate and mid-term results. The major neurologic morbidities and mortality compare favorably with CCE and are low.

Although, both stents achieved high packing densities, neither was the low recanalization rate nor the frequently observed progressive aneurysm occlusion affected by packing. Thus, hemodynamic flow changes facilitated by the placement of self-expandable stents may contribute to successful treatment and long lasting aneurysm occlusion as indicated for recently developed flow diverter stents.
